# End-stage kidney disease patients with severe coronavirus disease: clinical characteristics, biological data, and mortality in nephrology unit, short communication

**DOI:** 10.1097/MS9.0000000000000962

**Published:** 2023-06-28

**Authors:** Malika Ramdani, Hanae Oujidi, Hicham Elmaghraoui, Naima Abda, Yassamine Bentata

**Affiliations:** a Laboratory of Epidemiology, Clinical Research and Public Health, Faculty of Medicine Oujda; b Nephrology—Dialysis and Kidney Transplantation Unit, University Hospital Mohammed VI, University Mohammed Premier, Oujda, Morocco

**Keywords:** COVID-19, dialysis, end-stage kidney disease, lung damage, mortality

## Abstract

Patients suffering from end-stage kidney disease (ESKD) are particularly vulnerable to SARS-CoV-2 infection, and their risk of death is higher than for the general population. The objective was to determine the epidemiologic profile at admission and mortality among patients presenting EKSD with severe coronavirus disease (COVID-19).

**Methods:**

A retrospective study was conducted in the Nephrology unit between October 2020 and February 2022. Were included all adult patients who presented ESKD on dialysis, or not on dialysis with an estimated glomerular filtration rate less than or equal to 15 ml/min/1.73 m^2^ and presenting a confirmed COVID-19. Patients with ESKD who were immediately admitted to the ICU were excluded.

**Results:**

Sixty-five patients’ data were collected. The mean age was 58.9 ±16.7 years and 60% were males. Hypertension arterial and diabetes observed in 75% and 56.3% of cases, respectively. 52.3% were on haemodialysis, 4.6% were on peritoneal dialysis and 43.1% not were on dialysis. 94% of the patients were symptomatic of COVID-19, dominated by dyspnoea (87.5%), cough (65.6%), and fever (58.5%). More than half of patients (58.5%) showed signs of gravity and 62% required oxygen therapy. According to thoracic scan, 72.3% were classified COVID-19 Raw Data System 5 and 6. Most patients had severe anaemia (58.5%), lymphopenia (81.3%), and high levels of C-reactive protein (54%), D-Dimer (93.6%) and ferritin (91.2%). 38.5% of patients presented complications of whom 60% were transferred to ICU. Mortality was observed in 8% of cases.

**Conclusion:**

Rigorous monitoring is necessary for patients in ESKD, particularly those with comorbidities, to reduce the risk of severe form of COVID-19.

## Introduction

Patients suffering from end-stage kidney disease (ESKD), whether on dialysis or not, are particularly vulnerable to SARS-CoV-2 infection and their risk of death secondary to COVID-19 is higher than for the general population^1,2^. The analysis of Preliminary Medicare COVID-19 Data Snapshot noted that 2614 cases were found per 100 000 patients with ESKD compared with the general population rate of 518 cases per 100 000 that is a risk 4–5 times higher^3^. Note that this risk of COVID-19 infection was lower among ESKD patients on peritoneal dialysis than among ESKD patients on haemodialysis. In another large study, among 412 haemodialysis centres in 78 countries and all 10 ISN (International Society of Nephrology) regions, the average COVID-19 infection rate was around 20% with large variations observed between regions and countries^4^. In this study, rates of COVID-19 infection in staff ranged from 0 in 90% of centres in North East Asia to greater than or equal to 50% in 63% of centres in the Middle East and 68% of centres in Newly Independent States and Russia. According to a systematic review and a meta-analysis conducted among 568 533 patients on dialysis of which 10% were on peritoneal dialysis, the rate of COVID-19 hospitalization and mortality were higher with variations relating to ethnicity and dialysis modality. The lowest rates of COVID-19 hospitalization and mortality were observed in patients on peritoneal dialysis compared with patients on haemodialysis or kidney transplants^5^. For dialyzed patients with COVID-19, the reported mortality rate ranges from 10 to 30%, thus confirming the severity of the infection in this population^5–7^. In a multicenter study comparing mortality between four groups of patients [control vs. Haemodialysis vs. kidney transplant vs. chronic kidney disease (CKD)] the mortality rates were significantly lower in the control group (4%) than in the all-kidney disease groups (haemodialysis 16.2% and kidney transplant 11.1%), and higher in the CKD group (28.4%) than in the other groups^8^. In this study, the severity of the disease, biological inflammation and patient group (being in chronic hemodialysis or CKD groups) were significantly related to mortality. The multivariate analysis of 105 patients with stage 5 of CKD on dialysis identified as risk factors associated with mortality: severe COVID-19 disease, increased C-reactive protein, and decreased arterial oxygen pressure/inspired oxygen fraction^9^.

However, recent studies argue against this finding. In the first recent study, the authors found that without adjustment, ESKD patients hospitalized for COVID-19 had a significantly higher odds of in-hospital death than patients without ESKD, but once comprehensively adjusted, there was no excess risk of death for ESRD patients^10^. In the second study, the authors found that among individuals with COVID-19-positive test result, ESKD patients did not have a higher odd for adverse outcomes (mortality, stroke, shock, intubation, sepsis, pneumonia) compared with matched individuals without ESKD^11^. The objective of this study was to determine the clinical, biological, and radiological characteristics on admission and mortality in patients presenting ESKD with severe COVID-19.

## Materials and methods

This was a retrospective and observational single-centre study conducted in the Nephrology Department between October 2020 and February 2022. The month of October 2020 corresponds to the month of care for the first COVID-19 patients with an ESKD at our nephrology unit. The month February 2022 corresponds to the end of the third and last major national wave of COVID-19. Patients included in the study were all those who presented at admission ESKD with a glomerular filtration rate lower or equal to 15 ml/min/1.73 m^2^ not yet on dialysis, or on haemodialysis, or on peritoneal dialysis, and presenting a confirmed COVID-19 infection. Glomerular filtration rate was estimated using the Modification of Diet in Renal Disease (MDRD) formula. COVID-19 cases were retained based on a positive Reverse Transcriptase-Polymerase Chain Reaction (RT-PCR) or on clinical (fever, cough) and biological (elevated blood ferritin, blood D-dimer and C-reactive protein with lymphopenia) and radiological criteria [COVID-19 Raw Data System (CO-RADS) > 4] Certain included patients had previously been followed in nephrology with a known diagnosis of ESKD. In case of no previous care in nephrology, the chronic and terminal characteristics of kidney disease were retained retrospectively based on clinical, laboratory and radiological criteria. Patients with ESKD who were immediately admitted to the ICU for a severe form of SARS-COV-2 infection and kidney transplant patients were excluded. Patient data were mainly collected from computerized patient records including clinical and biological and radiological data. However, to have additional information for some patients, we also consulted the medical files in paper version. All statistical calculations were performed using the Statistical Package for the Social Sciences software version 21.0. Quantitative variables were expressed as mean ± standard deviation or as medians and inter-quartile range, depending on their distribution.

## Results

During the study period, 65 patients meeting the inclusion criteria were collected. Among them, 43.1% of patients not were on dialysis, 52.3% of patients were on haemodialysis and 4.6% were on peritoneal dialysis. The median duration of dialysis for patient under haemodialysis and peritoneal dialysis was 24 (12.48) months. The mean age of all included patients was 58.9±16.7 years, with extremes ranging from 16 to 98, 81.5% of patients were over 50 years old and 13.8% of patients were over 75 years old. The evolution of the number of confirmed COVID-19 cases showed three peaks during the study period: November 2020 (*n*=9), August 2021 (*n*=9), and January 2022 (*n*=7), consistent with the national trend of the infection. Real-time-PCR of SARS-COV-2 was positive in 80% of patients and 24% of them were already in contact with a COVID-19-positive person in their close entourage. Upon admission at the hospital, 58.7% of patients presented signs of gravity and 61.2% required oxygen therapy. Before their admission in our unit, 53.5% of patients had already visited a health facility and 78.5% of patients had physical autonomy. The average oxygen saturation was 86.3±10.3% and 60.9% of patients had a saturation of less than to 92%. Oligo-anuria (<400 ml/day) was noted in 52.4% of cases. Concerning biological data, 89.1% of patients had a blood haemoglobin less than11 g/l and 58.5% had a blood haemoglobin less than 8 g/dl, 80% of patients had lymphopenia, 54% of patients had C-reactive protein greater than 100 mg/l, 48.4% of patients had neutropenia (<1500 elements/mm^3^), 93.6% of patients had high blood D-Dimer (>0.5 mg/l), 91.2% of patients had high blood ferritin (>280 ng/ml), 96.4% of patients had high blood lactates dehydrogenase (>220UI/l), 89.1% of patients had high blood fibrinogen greater than 4 ng/l, 81.5% of patients had high blood troponin and 48.4% of patients had hypocalcemia (<80 mg/l). Thoracic scan was performed in 92% of cases and showed in 85% of cases typical images of COVID-19. COVID-19 Raw Data System (CO-RADS) stages 5 or 6 were observed in 72.3% of cases and CO-RADS stages 3 or 4 were observed in 27.7% of cases. In effect, 87.5% had an alveolar-interstitial syndrome. Pleural effusion was confirmed in 20% of patients and one patient had a pulmonary embolism. Table 1 reported the sociodemographic, clinical, and biological characteristics and complications that occurred in hospitalized patients with ESRD and COVID-19 in the nephrology department.

**Table 1:** Sociodemographic, clinical and biological data and complication occurred of patients hospitalized for ESKD with COVID-19

Parameters (*N*= 65)	All patients, *N* (%)
Mean age, years^a^	58.9±16.7
Elderly patients (age > 65 years)	22 (33.8)
Male sex	39 (60)
Comorbidities	
Arterial hypertension	48 (75)
Diabetes	36 (56.3)
Heart disease	9 (14.1)
Tobacco use	8 (13.1)
Dialysis modalities	
Chronic haemodialysis	34 (52.3)
Peritoneal dialysis	3 (4.6)
Admission clinical symptoms	
Fever	59 (93.7)
Cough	38 (58.5)
Dyspnoea	42 (65.6)
Chest pain	56 (87.5)
Nausea and/or vomiting	24 (37.5)
Diarrhoea	20 (31.3)
Others	14 (21.8)
Blood creatinine, mg/dl^a^	11.2±4.8
Blood urea, g/l^a^	2.23±0.83
Blood haemoglobin, g/dl^a^	8.11±2.35
Blood white cells, cells /mm^3^ ^b^	9785 (6652–12917)
Blood C- reactive protein, mg/l^b^	114 (56–199)
Blood procalcitonin, ng/ml^b^	2.32 (0.94–12)
Blood calcium, mg/l^a^	83.0±10.6
Blood phosphorus, mg/l^a^	68.9±19.4
Blood potassium, meq/l^a^	4.9±1.2
Blood albumin, g/l^a^	32.3±4.6
D-dimers, mg/l^b^	2.87 (1.13–6.05)
Blood ferritin, ng/ml^b^	920 (505.5–1500.6)
Complication occurred	25 (38.5)
Respiratory distress	14 (56)
Severe sepsis	5 (20)
Pericarditis	2 (8)
Gastrointestinal bleeding	1 (4)
Kidney replacement therapy	53 (81.5)
Indication for hemodialysis	
Uraemic syndrome	29 (44.7)
Acute lung oedema	14 (21.6)
Hyperkaliemia	11 (16.9)
Anaemia required blood transfusion	6 (9.2)
Oligo-anuria (<500 ml/day)	5 (7.7)
Transfer to ICU	15 (23)
Intrahospital mortality	4 (8)

a Variables expressed as mean, SD.

b Variables expressed as median, quartiles.

ESKD, end-stage kidney disease.

Regarding treatment, azithromycin was used in 83.5% of cases whereas Hydroxychloroquine and anti-interleukin 6 have only been used for four and one patient, respectively. Emergency dialysis was initiated in 83% of patients. The vascular access used was mainly a temporary catheter (jugular or femoral) in 67.7% of cases, a native arteriovenous fistula in 26% of cases, and a tunnelled catheter in 6.2% of cases. Complications had occurred in 38.5% of patients of whom 60% were transferred to the ICU, essentially for respiratory distress. Among patients not transferred to the ICU, and who remained hospitalized in the nephrology unit, the mortality was 8% (*n*=4). Three deceased patients were on haemodialysis. For deceased patients, the average duration of ESKD was 42±12 months, all patients had arterial hypertension, two patients had diabetes and three patients had anaemia and lymphopenia. All deceased patients had elevated levels of high C-reactive protein, procalcitonin, fibrinogenemia, ferritinemia, LDH, and troponin. CO-RADS 4 was observed in one patient and CO-RADS 5 was observed in three patients. Three deaths were secondary to acute respiratory distress syndrome and one death was secondary to severe sepsis.

## Discussion

The mean age of our patients was 58.9±16.7 years. Our patients are relatively young compared with the average age reported by other studies^2,5,12^. Most of our patients had at least one associated comorbidity. In other studies, arterial hypertension and diabetes have been reported in proportions ranging from 48 to 90% of cases^2,5,12^. The proportion of tobacco users among our patients was low (13.1%) compared with 26.4% reported in the Lebanese series of Aoun *et al.* ^12^, . On admission, 94% of our patients were symptomatic. The predominant respiratory symptom was dyspnoea (87.5%) which was more striking compared with findings in the other studies^2,12^. In our study, 58.5% of patients had fever at admission. In contrast, in other series, over half of the patients were fever-free^13^. At admission, a minority of our patients (6%) were asymptomatic of COVID-19. In the other studies, the prevalence of asymptomatic patients was relatively higher and reached 20%^1,12^.

This significantly increases the risk of infectious clusters within dialysis centres and households and makes it difficult to identify and prevent infection. Hence, the importance of screening all dialyzed patients even without clinical signs. In our study, 20% of patients had a negative RT-PCR, 46.5% had not visited a health facility before their hospital admission, and only 24% of cases had been in contact with a person positive for SARS-CoV-2. The latter finding was contrary to that in Wuhan, where this feature of contact was more marked and reached 48%^1^. These findings emphasize that a negative RT-PCR test in a dialyzed patients without known contact with a person infected with COVID-19 in their entourage, does not exclude the presence of SARS-COV-2 in this high-risk population. Consequently, additional diagnostic investigations are necessary, notably laboratory and radiologic examinations, to confirm or rule out SARS-COV-2 infection among patients suffering from ESKD. The majority of patients were anaemic due to inadequate erythropoietin production and had high levels of inflammation markers. Thus, rigorous monitoring of inflammatory parameters in ESKD patients with SARS-COV-2 appears to be indispensable to allowing early identification of patients with a poor prognosis. Most patients were put on Azithromycin. Hydroxychloroquine was rarely prescribed, contrary to data in other series 80% in Italy, 58% in New York^2,4^. Note that our patients were treated according to the national protocol for severe COVID-19 forms developed by our Ministry of Health.

The intrahospital mortality rate, in nephrology unit, was 8% and remains relatively higher. Note that our nephrology unit had been caring for serious patients (as evidenced by the admission parameters) because all beds in the ICUs were occupied during the three peaks of the three major waves of COVID-19. In addition, 23% of our patients were transferred, following a worsening of their clinical condition, particularly respiratory, to an ICU. Overall mortality including ICUs would thus be higher. Previous studies had described higher mortality rates ranging from 22 to 40%^1,2,12,13^. This mortality rate was higher in patients on haemodialysis than in patients on peritoneal dialysis^1,14^. In our series, the two main causes of death were hypoxia and severe sepsis. Fontana *et a*l.^2^ reported that respiratory failure was the main cause of death followed by sepsis. In the large series of Haaehaus *et al*.^1^, the main cause of death in chronic haemodialysis patients was infection, observed in more than 90% of cases.

All of our deceased patients had high C-reactive protein and D-dimer with features on the thoracic scan classified CO-RAS 4 or 5. Previous studies had clearly shown the association between death, comorbidities (such as arterial hypertension, diabetes, cardiopathy, obesity, etc.) and increased inflammatory parameters in chronic haemodialysis patients. The main independent risk factors for mortality in chronic haemodialysis patients were advanced age (>70 years old), male gender, ischaemic heart disease and long duration of dialysis^2,3^. The presence of an arteriovenous fistula was associated with improved survival, compared with other types of dialysis vascular access^1^.

In addition, the COVID-19 pandemic has led to significant changes in the organization of healthcare structures around the world. In our country, public healthcare structures are organized into three levels, level I (primary care), level II (secondary care) and level III (tertiary care). University hospitals, like our establishment, belong to level III and only treat patients with severe pathologies and/or associated severe comorbidities (diabetes, arterial hypertension, heart disease, chronic respiratory pathology, obesity, etc.). The capacities of the ICUs were quickly exceeded, forcing many other hospital departments such as the nephrology department to take care of patients in relatively serious condition, thus explaining the profile of the patients included in our study. We also carried out a reorganization of the acute dialysis units with maintenance of the usual nephrological activities^15^. While patients with less severe COVID-19 were cared for in level II care structures (non-university hospitals). This overall organization was satisfactory despite the difficulties encountered such as the insufficient number of nursing staff and the lack of certain equipment and consumables.

Our study provides interesting data even if the sample was relatively small. A larger cohort of ESKD patients hospitalized for COVID-19 would have provided more precise and relevant data.

## Conclusion

Our study showed that COVID-19 patients with ESKD, a particularly vulnerable population, had a higher risk of symptomatic severe disease and mortality. These results bring out the need to strengthen measures of COVID-19 prevention and monitoring for all ESKD patients whether they are on dialysis or not. Although our study has added to knowledge about the epidemiology of COVID-19 among patients with ESKD at our department, other prospective studies will be needed to determine more accurate data. Both diagnostic and therapeutic management must be rapid and adapted for a better prognosis of these patients without forgetting the major role of vaccination against SARS-COV-2.

## Ethical approval

This work does not contain any personal images or figures. Due to the observational and retrospective nature of the study and the COVID-19 period, recourse to the ethics committee was not necessary. This study was performed in absolute respect of the international ethical rules, anonymity, and data protection.

## Consent

Consent was not requested as the study was retrospective and observational.

## Source of funding

This work has not received any financing or sponsorships.

## Author contribution

M.R., H.O., H.E., N.A., Y.B., M.R., H.O. and H.E.M.: collected all patient data. Y.B.: designed the study and wrote the manuscript. N.A.: made statical analysis. All authors read and approved the final manuscript.

## Conflicts of interest disclosure

The authors declare that they have no conflicts of interest.

## Research registration unique identifying number (UIN)

My study is retrospective and observational.

## Guarantor

Yassamine Bentata.

## Data availability statement

The datasets used and/or analysed during the current study are available from the corresponding author on reasonable request. The file is available in SPSS file format including the data from 65 patients.

## Provenance and peer review

Not commissioned, externally peer-reviewed.
